# Microfluidics for interrogating live intact tissues

**DOI:** 10.1038/s41378-020-0164-0

**Published:** 2020-08-24

**Authors:** Lisa F. Horowitz, Adán D. Rodriguez, Tyler Ray, Albert Folch

**Affiliations:** 1 Department of Bioengineering, University of Washington, Seattle, WA 98195 USA; 2 Department of Mechanical Engineering, University of Hawaiʻi at Mānoa, Honolulu, HI 96822 USA

**Keywords:** Engineering, Materials science

## Abstract

The intricate microarchitecture of tissues – the “tissue microenvironment” – is a strong determinant of tissue function. Microfluidics offers an invaluable tool to precisely stimulate, manipulate, and analyze the tissue microenvironment in live tissues and engineer mass transport around and into small tissue volumes. Such control is critical in clinical studies, especially where tissue samples are scarce, in analytical sensors, where testing smaller amounts of analytes results in faster, more portable sensors, and in biological experiments, where accurate control of the cellular microenvironment is needed. Microfluidics also provides inexpensive multiplexing strategies to address the pressing need to test large quantities of drugs and reagents on a single biopsy specimen, increasing testing accuracy, relevance, and speed while reducing overall diagnostic cost. Here, we review the use of microfluidics to study the physiology and pathophysiology of intact live tissues at sub-millimeter scales. We categorize uses as either in vitro studies – where a piece of an organism must be excised and introduced into the microfluidic device – or in vivo studies – where whole organisms are small enough to be introduced into microchannels or where a microfluidic device is interfaced with a live tissue surface (e.g. the skin or inside an internal organ or tumor) that forms part of an animal larger than the device. These microfluidic systems promise to deliver functional measurements obtained directly on intact tissue – such as the response of tissue to drugs or the analysis of tissue secretions – that cannot be obtained otherwise.

## Microengineering: Microfluidic systems for physiological study of live, intact tissues

Microfluidics constitutes an invaluable tool for precisely stimulating, manipulating, and analyzing the intricate microarchitecture in live tissues and optimizing mass transport. Microfluidic systems are becoming an essential tool for studying intact tissues with in vitro and, increasingly, in vivo applications. A team headed by Albert Folch at the University of Washington, USA conducted a review of the use of microfluidics to study the physiology and pathophysiology of live, intact tissues at sub-millimeter scales. These studies show that microfluidic applications to intact systems have enabled more sophisticated study of animal and plant development, physiology, and pathophysiology, as well as drug delivery, to different tissues in larger animals, including humans. They believe that the wide-ranging applications of microfluidics to intact tissues have considerable future potential in research and health care.

## Introduction

Biologists have long recognized that tissue dissociation breaks apart essential components of the tissue architecture. These components, such as the vasculature, cell–cell neighbor interactions, and signaling gradients, play key roles in the organism physiology over the whole range of spatial scales – from the molecular and cellular scale to the organ and whole-organism scale. The German biologist and 1931 Nobel laureate Otto Warburg, a pioneer in the study of tissue metabolism, introduced the tissue slicing technique in 1923 as an attempt to preserve aspects of the tissue microenvironment and tissue function of tumors in vitro^1^.

The tissue slicing technique caught the attention of biologists working on two organs whose functions are most critically dependent on tissue architecture and its underlying cell–cell interactions: the brain and the liver. Henry McIlwain^2^ was able to prepare viable brain slices and published in 1966 a seminal electrophysiology paper demonstrating, for the first time, synaptic transmission in slices^3^. Klaus Brendel and Carlos Krumdieck (the developer of the Krumdieck slicer^4^) perfused thin tissue slices of the liver^5,6^ to show improved functionality with respect to dissociated cultures.

Brendel’s “precision-cut tissue slicing” technique was further expanded to many organ systems. The Brendel–Krumdieck team subsequently pioneered sliced cultures of other organs such as kidney^7^, lung^8^, heart^9^, and prostate^10^. The technique is now used in thousands of laboratories worldwide, and a multitude of models of tissue slicers – with options of microtome setting, vibrating blade, cooling, etc. – are now commercially available. In the last decade or so, when the role of the tumor microenvironment emerged as a key factor in the progression of cancer^11^, cancer biologists started using tumor slices with success^12,13^.

In combination with these precision-slicing techniques, other efforts were directed towards the development of “organotypic culture” techniques aimed at preserving tissue architecture and function^14^. In many of these first studies, the slices were completely submerged in fluid, which presented a challenge for proper oxygenation of the tissue due to the reduced diffusion of oxygen through aqueous fluids. Perfusion chamber designs based on perfusing slices atop a nylon mesh were initially proposed to enhance the longevity of acute slice preparations^15,16^. Haas et al.^15^ presented a design (known as “the Haas interface chamber”) where slices were in contact with a humidified air interface and placed atop a nylon mesh that was used to gently transport a flow of nutrients under the slices. To improve oxygenation, Stoppini et al.^17^ introduced a non-perfusion technique that keeps brain slices on top of a porous membrane and in contact with humidified air; the porous membrane allows for nutrient delivery to the basal side of the slice by diffusion. The porous membranes are now commercially available in various user-friendly formats and porosities (e.g. Transwell^TM^).

Microfluidics has revolutionized biomolecular and cell analysis, impacting fields ranging from biophysics to biochemistry and from cell biology to clinical medicine^18^. These devices can offer several advantages such as low reagent consumption, fast response time, low fabrication time, control of microscale cellular interactions, and nanoscale mass transport, in a compact package that provides scalability and user-friendliness^19,20^. Microfluidic systems are becoming an essential tool for addressing intact tissues. A recent review has focused on ex vivo systems^21^, referring to systems for studying primary tissues or cells in vitro, but microfluidic technologies historically developed for in vitro applications are now increasingly adapted to in vivo applications. Intact tissues are usually subjected to a surface constraint (i.e. in ex vivo systems where the tissue is scarce) or a volume constraint (i.e. in in vivo systems where the tissue is surrounded by other live tissues) that makes them ideally suited for microfluidics. Here we define “intact tissues” as an ensemble of cells whose 3D biomolecular and cellular architecture is preserved throughout experimentation, in short, one that has never been dissociated into smaller entities during their lifetime; it is understood that the tissue – whether in culture or as a result of in vivo manipulation – might incur some cell death, or loss of cell phenotype, altering the natural relationships seen in intact tissues, but the ultimate goal of the experimental technique should be to avoid those functional losses. This review covers (1) the plethora of in vitro studies where a tissue explant is introduced inside the microfluidic device for manipulation and analysis (with contributions grouped by scientific field of research); (2) in vivo studies where a whole organism is small enough to be introduced into a microfluidic device; (3) in vivo studies where a microfluidic device is used to sample and analyze fluids from the skin’s surface (i.e. sweat; note that saliva and tear fluid micro-analyzers, while conceptually similar, are not considered because the tissue itself does not typically come into contact with the device); and (4) in vivo studies where a microfluidic device performs localized drug delivery after placement partially or fully inside the human or animal body.

## In vitro studies

As compared to traditional fluid delivery methods, microfluidic delivery to intact tissues provides a more precise design of the cellular microenvironment, a higher reproducibility of the fluid delivery, a more quantitative approach to mass transport, and a multiplexing approach that is more efficient in terms of real estate, delivery time, and/or reagent consumption. These microfluidic devices have been used to miniaturize the petri dish, i.e. recreate cellular microenvironments in vitro, as well as to miniaturize the clinic, i.e. recreate and multiplex the application of drugs to a patient’s cells in vitro or to an animal model^20^. First we will discuss microfluidic studies in humans and other animals, then turn to studies in plants.

### Development

**Fig. 1:**
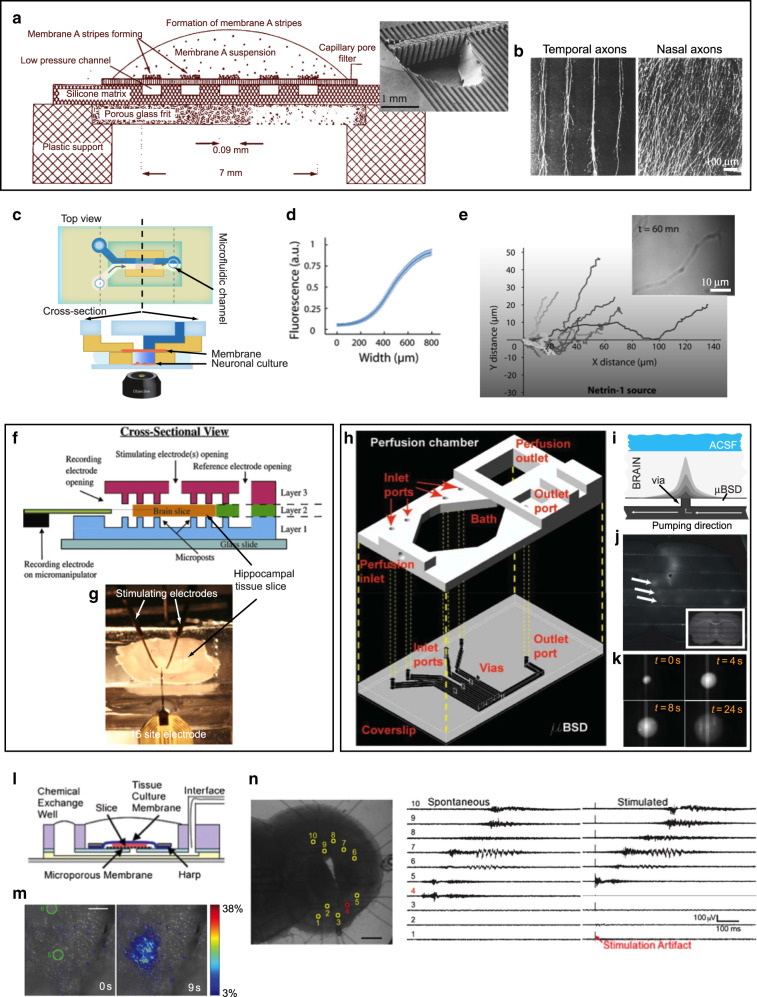
Microfluidic interrogation of intact neural tissues. **a**, **b** “Stripe assay” for axon guidance. **a** Cross-sectional schematic of a porous membrane assembled atop a set of micromolded PDMS channels; application of negative pressure to the microchannels with fluid on top of the porous membrane causes hydrodynamic focusing of flow of particulates in suspension (such as cell membrane fragments) towards the stripes defined by the microchannels. Inset: scanning electron micrograph of the PDMS device prior to assembly with the membrane; a hole has been made to reveal the set of vertical channels that provide fluidic access to the horizontal channels. **b** Optical image of the porous membrane area after a retinal explant is allowed to grow at the edge; the growth of temporal axons is clearly guided by stripes created with cells from the anterior tectum (left), whereas stripes created with cells from the posterior tectum do not guide their growth (right). Adapted with permission from ref. ^22^. **c**–**e** A microfluidic assay for amplification and temporal filtering during gradient sensing by nerve growth cones. **c** Top and cross-sectional view of the Y-shaped microfluidic device used in the study. A fluidic microcircuit is interfaced via a porous membrane with neuronal cultures in a microwell. Co-flow in the micro-circuit generates a shear-free gradient in the microwell. **d** The concentration profile at the coverslip surface, measured by confocal microscopy and obtained by averaging profiles measured every 30 s over 1 h. The fluctuations of the relative gradient in the central part of the device are less than 5%. **e** Example of trajectories of individual growth cones in the Netrin-1 gradient. Inset: Turning and elongation of an axon in the netrin-1 gradient. Adapted with permission from ref. ^28^. **f**, **g** PDMS microfluidic perfusion chamber for electrophysiology. **f** Cross-section of the device. **f** Photograph of the setup, showing the stimulating and recording electrodes. Adapted with permission from ref. ^33^. **h**–**k** Microfluidic add-on for the standard electrophysiology chamber setup. **h** CAD drawing showing how the microfluidic channels are added below the electrophysiology chamber. **i** Schematic representation of fluid delivery by one of the channels to a brain slice. **j** Fluorescence micrograph showing a mouse brain slice with delivery of FITC (1 µL dispensed at the inlet port) in spots as indicated by the three white arrows. The inset is a brightfield image of the same slice. **k** Montage of fluorescence images (taken at various intervals) showing FITC (1 µL dispensed at the inlet port) in channels and delivered to the slice from below. Adapted with permission from ref. ^35^. **l**–**n** Microfluidic microelectrode array. **l** Cross-section schematic of the device. **m** Pseudo-colored fluorescence micrograph demonstrating induced local activity (as visualized by calcium imaging) at 9 s after low amplitude electrical stimulation in the spots marked with green (0 s). **n** Electrical traces (right) of spontaneous and stimulated waves recorded from a slice (right). Adapted with permission from ref. ^37^

The first microfluidic studies with live tissue explants were pioneered by the Bonhoeffer group in 1987^22^. They devised an ingenious PDMS-based microfluidic device for immobilizing two types of cell membrane fragments (Fig. 1a) that served as molecular guidance cues for the growth of neurons from retinal explants (Fig. 1b). The device was composed of microchannels molded in PDMS from a photolithographically etched master. A porous membrane (a Nucleopore filter with pore size 0.1 µm) was placed atop the microchannels. When a suspension of cell membrane debris was allowed to flow on top of the device and, simultaneously, suction was applied through the microchannels, the cell membrane debris were hydrodynamically trapped on the top surface of the filter (Fig. 1a). Once the filter got clogged and stripes of the membrane were formed, the filter was moved to the top of another porous membrane matrix that had homogeneous porosity (not containing microchannels) and a different cell extract was added on top of the filter. When suction was re-started, the membrane fragments from this new cell extract got immobilized in the areas between the old stripes, forming stripes of two different kinds. With this micropatterning technique, the researchers were able to confirm the hypothesis that specific molecules present in the membrane of cells from selective regions from an optic tectum explant of a chick could differentially guide the growth of retinal axons from another explant. They observed that temporal axons showed a preference for growth on membranes of the anterior tectum (their natural target area) over posterior tectum, whereas nasal axons did not show a preference (Fig. 1b).

For decades, developmental neurobiologists have used this setup, which became known as the “stripe assay”, to micropattern axons. In a simplification of the “stripe assay”, Bonhoeffer and colleagues showed that the porous membrane was not needed^23^. Drescher’s group used the PDMS channels to directly deposit the membrane fragments onto a plastic surface that had been pre-coated with nitrocellulose^24^. By placing explants onto the microfluidically patterned substrates, they observed that overexpression of ephrinA ligands on temporal axons abolished the selectivity previously observed by Bonhoeffer’s group, whereas treatment with PI-PLC both removed ephrinA ligands from retinal axons and induced a striped outgrowth of formerly non-selective nasal axons. This work suggested that differential ligand expression on retinal ephrinA5 axons is a strong determinant of topographic targeting in the projection of retinotectal axons.

Although the stripe assay was created with a microfluidic device, in the above studies microfluidics were not directly used to maintain the explants alive or to expose the axons to soluble cues. While many groups devised microfluidic devices to expose dissociated neurons to soluble axon guidance signals (see review of biomolecular gradients in cell culture^25^), probing explant cultures with microfluidics has been more challenging. Asbeck’s group designed a simple “alternate-choice” perfusion chamber for explants^26^. Here a laminar flow of two streams “forced” the growing axons from the explant to choose the attractive-factor stream. When rat spiral ganglion explants (300 µm × 300 µm in size) were placed in the tissue culture well, neurites preferentially grew towards the stream containing neurotrophin-3 (NT-3) as opposed to the stream without NT-3. A team led by Studer and Dahan devised a clever variation of the Bonhoeffer device; the channels were also placed facing up and closed with a porous membrane that served as the substrate of a cell culture chamber containing the axons, which grew from an adjacent well where the explants were introduced^27^. However, by flowing two different solutions (source and drain) in two microchannels separated by 200 µm, they were able to create a dynamic, shear-free gradient of the source molecules in the cell culture chamber above. At subthreshold repulsive levels, Slit1 acted as a potent promoter of both Netrin-1 attractive and repulsive activities on the growth cones of thalamic neurons from rostral thalamic explants. The same group created an inverted version of this device where closed microchannels featuring a porous membrane floor were placed atop an open tissue culture chamber (Fig. 1c), and were used to deliver soluble netrin-1 gradients to the growth cones of *Xenopus* spinal cord explant neurons below^28^ (Fig. 1d, e).

Like axon guidance, other processes such as neuronal migration and innervation play key roles in neural development. A team led by Irimia and Breakefield has addressed the challenge of measuring neuronal migration in embryonic mouse brain explants using microfluidics. After transfection of the explants with vectors to make them express GFP, the explants were placed in a chamber that communicated with microchannels, such that the migration of individual GFP-labeled neurons out of the explants (and into the microchannels) could be easily visualized and quantified^29^. Similarly, the Perlson lab placed mouse spinal cord explants in a chamber next to a set of microchannels with which they guided spinal cord neurons to grow towards and then innervate myotubes derived from muscle satellite progenitor cells^30^.

Microfluidics can also be harnessed to build simple perfusion interfaces with very small embryonic tissues. Nelson et al. built a microfluidic chamber with a catheter where they were able to intubate an embryonic lung explant. This device allowed them to visualize airway branching in time-lapse and simultaneously measure transmural pressure, which led them to conclude that transmural pressure controls airway branching morphogenesis, the frequency of airway smooth muscle contraction, and the rate of developmental maturation of the lung^31^.

### Neurophysiology

Microfluidics has a long history of applications to enhance brain electrophysiology. In traditional slice electrophysiology, a brain slice is typically exposed to a homogeneous bath solution, either static or dynamically exchanged by means of a perfusion chamber. To improve mass transport under the slice with respect to previous mesh substrates^15,16^, Passeraub et al.^32^ developed a microfluidic chamber where the slices were placed atop microengineered posts with defined spacing. However, exposure of the slice to a heterogeneous bath (e.g. one half of the slice to one drug and the other half to another) was challenging. Williams and colleagues built PDMS microfluidic perfusion chambers for electrophysiology that delivered heterogeneous laminar flows onto the surface of medullary brain slices (~530–700-µm-thick) from P0–P4 neonatal rats, so that the slices were locally perfused in “liquid stripes” of different biochemical environments^33,34^ (Fig. 1f, g). The design achieved independent control of fluids through multiple channels in two separate fluid chambers, one above and one below the slice, by setting the slice on top of microposts. Simultaneous electrophysiological recordings were achieved from the edge of hippocampal slices while the biochemical environment was modulated. Eddington’s group developed a microfluidic add-on for standard electrophysiology perfusion chambers that facilitated the multiplexing of fluids^35^ (Fig. 1h–k). Tang et al. developed a device for focal stimulation of brain slices based on placing the slice atop a “microfluidic fountain” that was composed of a central injector and surrounding suction ports; focal delivery of potassium chloride solution induced cortical spreading depression in mice brain slices^36^.

In all the above designs, the slices were submerged in a perfusion chamber, which can become an issue for proper oxygenation of the brain tissue (usually cultured with an air interface). Folch and co-workers developed a microfluidic multi-electrode array (MMEA) capable of high-resolution extracellular recording from organotypic brain slices (i.e. cultured on a porous membrane)^37^ (Fig. 1l–n). The MMEA device (Fig. 1l) was optically compatible with calcium imaging at single-cell resolution (Fig. 1m). The MMEA device was used to record waves of spontaneous activity in developing cortical slices and to perform multi-site extracellular recordings during simultaneous calcium imaging of activity (Fig. 1n). The device helped reveal the existence of two distinct pacemakers for spontaneous waves of activity in the developing mouse cortex^38^. Microfluidic devices can be bonded/assembled on top^39^ or at the bottom^40,41^ of Transwell membranes to selectively deliver solutions to cells, and potentially in the future to tissues. Notably, the Potter group^42^ and others^43,44^ have presented microfluidic setups with semi-permeable membrane roofs that are capable of perfusing flow through thick brain slices (“interstitial perfusion”).

Microfluidic devices for the neurophysiological recording of other tissues have followed. The retina has attracted the attention of experimental neurobiologists for a long time because a retina explant can be straightforwardly removed intact, laid down flat, and kept alive for extended periods of culture in saline, during which it remains electrophysiologically and photoactively normal. Thus, electrophysiological recordings of retina explants cultured on top of microelectrodes are now commonplace^45,46^. These recordings, however, do not allow for microfluidic modulation of the extracellular milieu. The Xu lab integrated graphene field-effect transistors into a microfluidic chamber and used the device in combination with optical detection to investigate electrical signals in mouse retina^47^. Edd’s group developed a “retina-on-a-chip” PDMS device that delivered solutions through 100-µm-diam. holes to the plane of a retina explant^48^. Whole mice retina were maintained for 4 days with negative pressure applied to the PDMS holes; nutrients were supplied to the retina from the top surface by diffusion through an agar disk set atop the retina in order to keep the retina flat. Local staining with toluidine blue, local application of cholera toxin beta, and transient response to lipopolysaccharide in the retina were all demonstrated. Quero and co-workers created an “autonomous” microfluidic chamber for the culture of mouse retinal explants that incorporated gold microelectrodes, a microheater, and a thermistor (for temperature control), and allowed for optical stimulation and real-time monitoring; the conditions tested had a protective effect on photoreceptor cell death^49^. Such devices could also be applicable to recording and stimulation of other types of electrically excitable tissue, such as muscle. Greenman and colleagues developed a microfluidic device for the perfusion of rat or human heart tissues with real-time electrochemical monitoring of the release of reactive oxygen species^50^.

### Liver toxicology

Despite the long history of liver slice cultures, microfluidic studies based on intact liver tissue remain rare. A number of groups and companies have developed perfusion systems for toxicology using primary hepatocytes, but not intact tissue – e.g. the devices by Hurel, Nortis, CellAsic, etc. Various microfluidic efforts have focused on recreating the 3D architecture of the liver (such as incorporating sinusoids with endothelial-like barriers), incorporating non-parenchymal cells that are known to be essential for liver function, developing vascularized human liver organoids, and mimicking liver zonation (see review of liver “on-a-chip”^51^). It remains to be investigated whether these microfluidic advances can be adapted to perfusion of intact tissues.

**Fig. 2:**
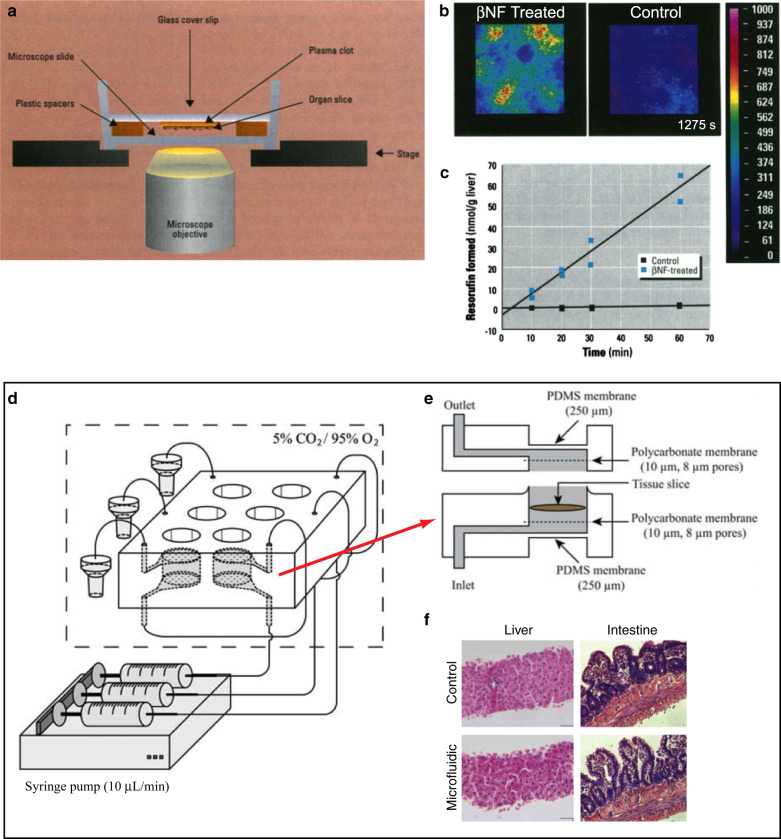
Microfluidic devices for liver toxicology using intact liver slices. **a**–**c** A perfusion chamber for liver slice perfusion. **a** Cross-section schematic of the setup on the microscope stage. **b** Confocal images showing a comparison of ethoxyresorufin-O-deethylase (EROD) activity in liver slices isolated from β-naphthoflavone (βNF)-treated and control rats. **c** A graph that compares the EROD activity in liver slices isolated from control (black squares) and βNF-treated rats (blue squares) in the perfusion cultures. Adapted with permission from ref. ^52^. **d**–**f** A microfluidic approach for in vitro assessment of inter-organ interactions in drug metabolism using intestinal and liver slices. **d** 3D schematic of the setup. **e** Cross-section schematic of the microfluidic device. **f** Histological evaluation of slices after 3 h of incubation in well plates (top), and in the microfluidic device (bottom). (Magnification: ×100). Adapted with permission from ref. ^56^

Kavanagh and colleagues published the first toxicology study of live tissue slices using a perfusion chamber^52^ (Fig. 2a). They used 250-µm-thick rat liver slices and confocal laser cytometry to determine the regional distribution of cytochrome P450 activities (Fig. 2b). The amount of resorufin (a fluorogenic substrate) formed was nearly 60-fold higher in slices treated with β-naphthoflavone (βNF, which induces the P450-dependent dealkylation of the resorufin substrate) compared to control slices (Fig. 2c). However, in their setup the slice was attached to a glass slide with plasma clot, so only one side of the slice was exposed to fresh nutrients (and thus created a gradient of nutrients in Z). Khong et al.^53^ built a miniature perfusion system in polycarbonate to perfuse 0.3–1-mm-thick precision-cut liver slices. To improve nutrient delivery, in addition to perfusion, they inserted into the tissue seven needles (300 mm O.D.) that directly delivered medium into the tissue slice. They observed stable Cytochrome P450–1A activity and an increase in the activity of uridine glucuronyl transferases for 3 days in the perfusion system.

A group led by Greenman and Haswell fabricated a simple Y-shaped microfluidic device in glass (by photolithography and wet etching) with channels of 190 µm width and 70 µm depth to perfuse previously cryopreserved liver biopsies (~4 mm^3^ sections)^54^. They manually placed the samples into the open-roof chamber (in a wider region of the channel) and then they manually closed the chamber’s roof by applying a plug. The eluent from both outlets of the microfluidic device was collected for 30 min and then combined for analysis. H&E staining revealed that the hepatocytes preserved their original hexagonal shape, and the tissue biopsy produced both albumin and urea for at least 70 h.

A team led by Groothuis and Verpoorte developed a PDMS microfluidic device with perfusion of precision-cut rat liver slices for liver toxicology^55^ (Fig. 2d, e). The group further used the device to assess inter-organ interactions with liver and intestinal slices^56^ (Fig. 2f). The slices were placed in adjacent microchambers and perfused sequentially so that the metabolites excreted by the intestinal slice were directed to the microchamber containing the liver slice. The morphology and metabolic rates of intestinal slices in the microfluidic device were similar to that in well plates. The interplay between the two organs was demonstrated by exposure of the slices to chenodeoxycholic acid (i.e. bile), which induced expression of fibroblast growth factor 15 (FGF-15) in the intestinal slice, and FGF-15, in turn, caused down-regulation of the detoxification enzyme cytochrome p450 activity in the liver slice.

### Cancer

Current methods to assess cancer treatments are often inaccurate, costly, and/or cumbersome. Recent reports on clinical drug development success rates indicate that 76% of clinical failures were due to a lack of efficacy (52%) or to safety issues (24%), with almost 30% being cancer drugs^57^, and that the average costs of developing a new cancer drug are now more than $650 million^58^. One of the main causes of this expensive gridlock is that pre-clinical animal tests do not accurately predict toxic doses and drug metabolism later observed in human trials^59^. Clearly, there is an urgent need for better functional drug assays based on human tissue which would more closely mimic patient disease and better predict traditional and immunotherapy responses.

Functional assays can potentially complement and extend genomics-based approaches to cancer drug development by capturing key determinants of therapeutic response such as tissue architecture, tumor heterogeneity, and the TME^60^. Diverse functional assay platforms have been developed that assess drug responses in tumor samples. The most acute challenge in functional precision cancer medicine arises from the fact that dissociated cells are insufficient for the functional assays – to preserve the TME, these assays should be performed on intact tissue, whose availability is scarce. The small sizes of the clinical samples has prompted many groups to use various approaches, including: (1) tumor spheroids (small spheres or “organoids” formed from patient-derived, dissociated cells), a model that can create cell–cell and cell–matrix 3-D interactions that more closely resemble in-vivo interactions and has been used for high-throughput drug screening assays^61^ that can be predictive of the patient’s responses^62,63^, but retains only a limited amount of the original TME because it relies on an amplification or growth step (see organoid review^64^); (2) patient-derived xenograft (PDX) mouse models that permit study of drug responses in an intact organism, including immune checkpoint blockade in humanized PDX^65^, but the rest of the TME is from the host mouse, and PDX from individual patients grow too slowly to inform initial post-operative therapeutic decisions; (3) tissue slices, a technique often based on culturing tissue slices atop a porous membrane support and recently applied to cancer slices with success^12,13^, but sensitive to tissue scarcity; (4) micro-dissected tumors (µDTs) based on sectioning of tumors into submillimeter tissue pieces that maintain the TME intact and are amenable to mass transport optimization and quantitative modeling^66–69^; and (5) implantable or needle microdelivery devices^70,71^ that locally deliver small doses of (up to 16) drugs to the tumor in vivo, with maximal preservation of the TME, but subject to limitations of tumor accessibility and patient safety. The first two approaches do not fit our definition of “intact tissue” so they are not reviewed below.

**Fig. 3:**
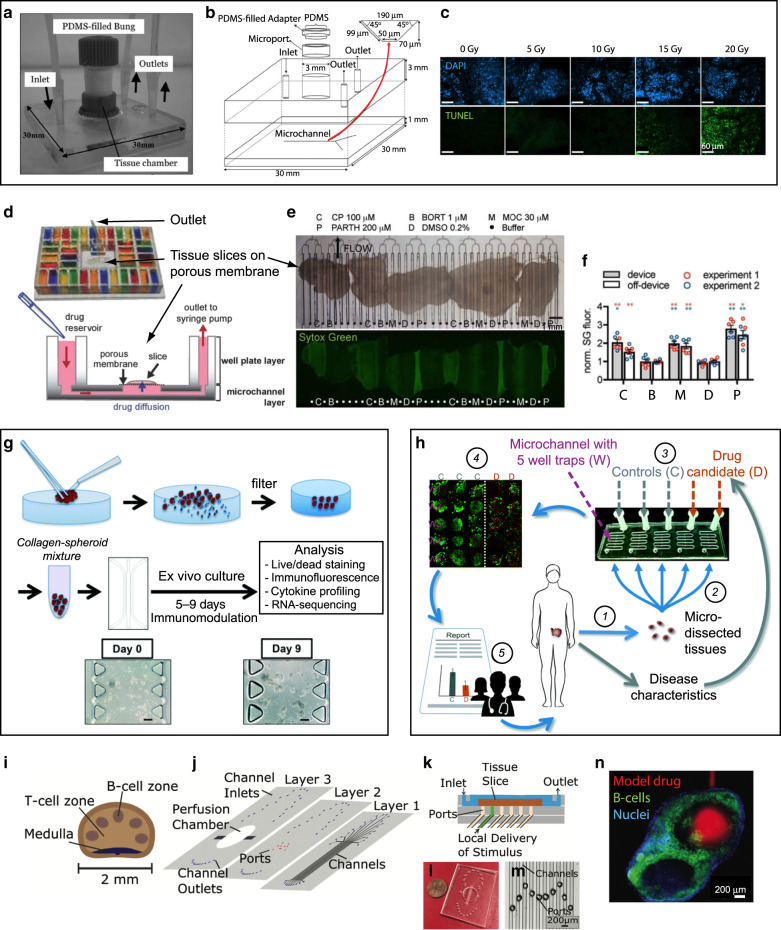
Microfluidic interrogation of cancer and lymphatic tissue. **a**–**c** A Y-shaped microchannel for measuring the responses of human head and neck squamous cell carcinoma biopsies to radiation. **a** Photograph of the device. **b** Schematic diagram of the device. **c** Micrographs showing DAPI (nuclei, top images) and TUNEL staining (DNA fragmentation after irradiation, bottom images) on lymph neck node metastases following microfluidic culture with control no irradiation (0 Gy) or different single-dose irradiations (5, 10, 15, and 20 Gy). Adapted with permission from ref. ^74^. **d**–**f** Microfluidic platform for drug sensitivity testing of intact tumor slices. **d** Photograph of the multi-well platform, with the inlet wells filled with food-coloring dyes (top); cross-section schematic of the device, showing a slice atop the porous membrane (bottom). **e** 48-h-long delivery of a panel of cancer drugs consisting of cisplatin (C or CP, 100 µM), bortezomib (B or BORT, 1 µM), mocetinostat (M or MOC, 30 µM), and parthenolide (P or PARTH, 200 µM), as well as a vehicle control (DMSO, 0.2%) and intervening buffer channels (•) to 5 U87 glioma xenograft flank tumor slices on the device. The top image is a brightfield micrograph at the beginning of the experiment, and the bottom image is a fluorescence micrograph after bathing the slices with SYTOX Green, a cell death indicator, at the end of the experiment. **f** The cell death readouts are consistent from experiment to experiment and comparable to off-device controls. Adapted with permission from ref. ^77^. **g** Workflow for drug treatment of micro-dissected tumors (µDTs), showing mincing, filtration, resuspension in collagen gel, culture in a central microchannel, drug treatment, and analysis. Delivery of immunomodulators is performed through the two side channels. Adapted with permission from ref. ^66^. **h** Workflow for drug treatment and analysis of µDTs placed into microfluidic PDMS traps. Each channel is perfused independently and contains 5 traps. Adapted with permission from ref. ^67^. **i**–**n** Microfluidic devices for probing lymph node slice cultures. **i** Schematic of the lymph node structure. **j** Exploded schematic showing the three layers of the device. **k** Cross-section schematic of the assembled device. **l** Top-view photograph of the device. **m** Micrograph of the center portion of the device containing a set of apertures for delivery of solutions to the tissue. **n** Local stimulation of the T-cell region of a lymph node slice with a model drug (Alexa Fluor-647-Glucose-BSA, red). To identify B-cell zones, live slices were also stained with FITC-anti-mouse B220 (green) and Hoechst (blue). Adapted with permission from ref. ^95^

Different groups have addressed the mass transport challenges that slices and biopsies face when placed in a microfluidic environment. A team led by Greenman and Haswell used their previously developed Y-shaped glass device for the interrogation of liver tissue biopsies^54^ to test the responses of human head and neck squamous cell carcinoma (HNSCC) tumor biopsies to the chemotherapy drugs cisplatin and 5-fluorouracil^72,73^. They found no loss of viability for 48 h and that the combination of both therapies was the most effective at causing cell death. A subsequent study measured the responses of human HNSCC biopsies to radiation^74^ (Fig. 3a–c). Folch and co-workers implemented the organotypic tissue slice technique in a microfluidic format to optimize tissue oxygenation/viability and be able to multiplex the delivery of drugs to brain and tumor slices from underneath a porous membrane^75,76^. The original microfluidic platform was PDMS-molded and is presently laser-cut in PMMA^77,78^ (Fig. 3d–f). The device allows for selective spatiotemporal exposure of organotypic cultures to dozens of drug conditions (Fig. 3d), and its utility has been demonstrated with cell death assays on xenograft (Fig. 3e) and patient slices^77,78^. With two types of cell death measures, comparable drug responses were shown between repeats on the device, and between on-device and off-device controls (Fig. 3f). Holton et al.^79^ developed a microfluidic device for the trapping and continuous perfusion of fine needle aspirate (FNA) biopsies. FNA biopsies of lung adenocarcinomas from mouse PDX were successfully trapped and treated with staurosporine as a proof of principle. The Greenman group recently used a milled polyether ether ketone (PEEK) plastic microfluidic device to culture human thyroid tissue slices (malignant and benign) and demonstrated tissue viability for >4 days using various assessments^80^. These microfluidic models could potentially be used to determine the response of an individual tumor to various chemotherapy and radiation regimens in vitro, prior to exposing the patient to the therapy and its devastating side effects, as well as aiding in the development of drugs that better treat solid tumors^81^.

Recent studies have applied microfluidic devices to micro-dissected tumors (µDTs) – small tumor pieces usually created by mincing with a scalpel – that attempt to preserve the TME. Similar to work from Kuo’s group, who re-suspended the µDTs in collagen gel and cultured them in Transwells^69,82,83^, a team led by Jenkins and Kamm also re-suspended the µDTs in collagen gel, but cultured them in microchannels^66,68^. They performed medium delivery and fluid recovery through connected, parallel channels, and demonstrated immune checkpoint inhibition in multiple cancer types (Fig. 3g). However, tissue mincing can suffer from serious shortcomings: (a) as a manual technique, it results in a large heterogeneity of µDT sizes; (b) size variability reduction by passage of the minced tumor through a set of filters, if performed, results in loss of precious tumor material; (c) expansion in culture, if done to obtain sufficient tumor material, necessarily alters the TME; and (d) as the µDTs are typically seeded at random, variability of tumor amount between different wells or chambers potentially confounds the results (e.g. in secretion assays). Using a mouse colon cancer model, Moore et al.^84^ loaded hand-selected tumor fragments from thawed needle cores into a 12-channel microfluidic device made of COC plastic; they then applied T cells and looked for cell death, including the effect of checkpoint inhibitor treatment. The Greenman group performed a large study (128 biopsies from 33 patients) in which they minced glioblastoma tumors into ~(2 mm)^3^ pieces; they manually introduced individual pieces into a simple Y-shaped microfluidic device that allowed for lactate dehydrogenate (viability) analysis and retrieval^85^. Gervais and colleagues prepared cylindrical µDTs of reproducible size by punching ~420 µm-diam. cores from slices from multiple types of xenograft tumors; the reproducible size of the µDTs allowed them to make a metabolite transport model, and they cultured the µDTs without hydrogel in a PDMS design containing 5 traps per microchannel^67^ (Fig. 3h). Eventually, for drug studies, PDMS designs need to be converted to drug-compatible materials; both absorption into PDMS^86–93^ and adsorption onto PDMS^94^ can potentially alter experimental outcomes by changing the effective drug concentrations and by partitioning molecules in undesired regions of a microfluidic device.

### Immunology

The lymph nodes are an important part of the body’s immune system; lymph nodes filter substances that travel through the lymphatic fluidic and contain lymphocytes that help the body fight infection and disease. The hundreds of lymph nodes in the human body are connected by lymphatic vessels that circulate the lymphatic fluid. The Pompano lab has developed microfluidic devices to selectively stimulate substructures within lymph node slices with submillimeter precision (Fig. 3i–n). One of the devices consisted of a PDMS perfusion chamber whose floor had a set of 10 holes, each of which could be addressed by a different microchannel^95^. When lymph node slices (Fig. 3i) were set atop the holes (Fig. 3j–m), they demonstrated the delivery of various solutions such as dyes (70 kDa FITC-dextran, 10 kDa Texas Red-Dextran), nuclear stain (Hoechst), or fluorescent antibodies (used to simulate immunotherapy drugs) (Fig. 3n).

A variation of this design utilized a two-component SlipChip^96^ to create a mobile port beneath the tissue slice in order to allow users to change the site(s) of stimulation^97^. The ports were laser-cut in acrylic layers. Using the same microchannel, but aligning the SlipChip with two different apertures, 40-kDa Dextran was delivered to stain two different locations of a lymph node slice. The researchers also produced a “dual slice” chip to study interactions between mouse lymph node slices and slices of a mouse model of breast cancer where tumors were grown for 7 days in the inguinal mammary fat pad^98^. The device maintained the two slices in separate chambers (each on a porous membrane) and re-circulated flow continuously. With the help of fluid dynamics simulations, they claimed that the flow passes transversely through the slices (although the possibility of leakage through the edges was not ruled out). Pompano’s devices could potentially be used to rationally design therapies for inflammatory disease.

### Endocrine system

The endocrine system is a tightly regulated network of glands that produces chemical messengers (called hormones). These hormones are released directly into the circulatory system and regulate metabolism, growth and development, tissue function, sexual function, reproduction, sleep, and mood, among other things. Many of these signaling events have been studied with microfluidics.

**Fig. 4:**
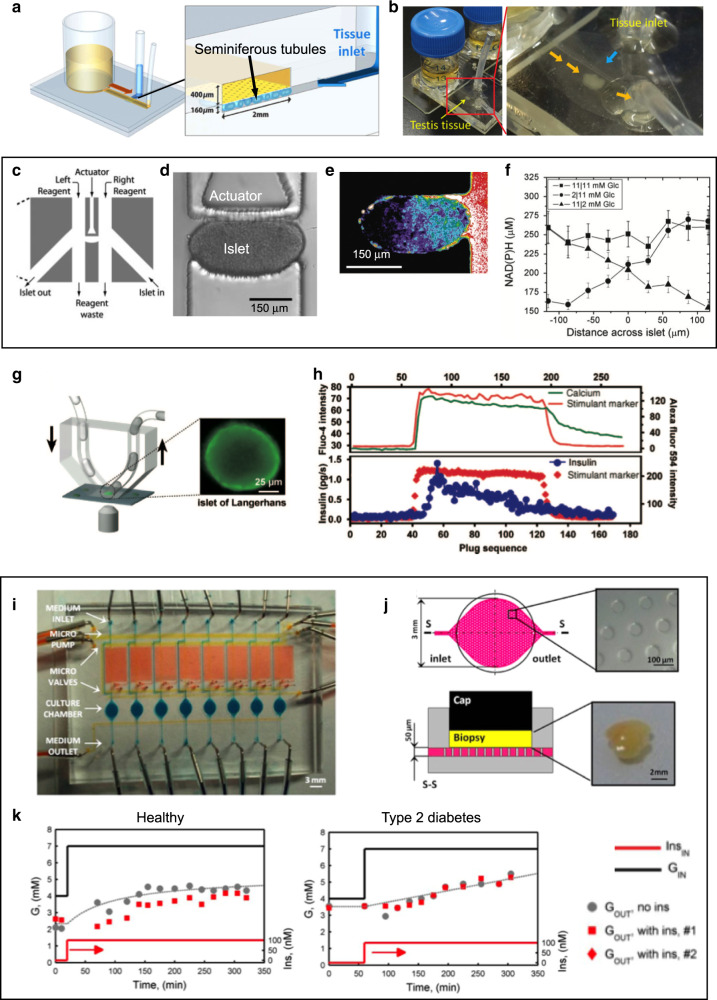
Microfluidics for interrogating endocrine tissue. **a**, **b** A microfluidic device for trapping and culturing seminiferous tubule tissue from mouse testes. **a** Schematic of the device; the inset shows a cross-sectional cut of the culture chamber, depicting the position of the porous membrane and the seminiferous tubules. **b** Top-view photographs of the whole device (left) and detail (right) showing the chamber where the testis tissue is trapped for culturing. Adapted with permission from ref. ^99^. **c**–**f** Microfluidic glucose stimulation of pancreatic islets. **c** Top-view schematic of the device design showing the actuator channel, the islet in and out channels, two reagent channels, and a reagent waste channel. **d** Islets are introduced by pressure-driven flow from the “in” channel and trapped by activation of the actuator channel. This actuation does not cause a NAD(P)H rise or internal [Ca^+2^] oscillations in the absence of glucose stimulation. **e** A representative islet after exposure to 2-NBDG (a fluorescent glucose analog) in the right stream. **f** Profile of the NAD(P)H concentrations across an islet exposed to 11/11, 2/11, and 11/2 mM glucose on the left/right side. Adapted with permission from ref. ^105^. **g**, **h** A droplet microfluidic setup for stimulating and recording from pancreatic islets with high temporal and chemical resolution. **g** Schematic of the setup (inset shows the micrograph of an islet). **h** Graphs showing the intracellular [Ca^+2^] response and insulin secretion of a stimulated mouse islet. The upper panel graph shows traces measured by fluorescence microscopy during stimulation (marker: Alexa Fluor 594, red) and recording (intracellular [Ca^+2^] by fluo-4 indicator, green). The lower panel graph shows traces of the fluorescence intensity of Alexa Fluor 594 marker and the calculated insulin secretion rate, measured with plugs collected during recording. Adapted with permission from ref. ^107^. **i**–**k** A microfluidic device to assess physiological responses of adipose tissue. **i** Photograph and layout of the device. **j** Top-view and cross-sectional view for schematic and images over the culture area. **k** Graphs showing the glucose response to insulin present in normal, but not in diabetic, adipose tissue. Adapted with permission from ref. ^118^

Fertility researchers have become interested in microfluidics because these systems provide exquisite control over the biophysical and biochemical environment of delicate tissue cultures. The Ogawa lab devised a microfluidic device with a 160-µm-tall chamber where mouse testis tissue could be introduced and separated from flow (in a separate channel) by a porous membrane^99^ (Fig. 4a, b). The testis consists of long convoluted tubular structures called seminiferous tubules, where spermatogenesis occurs. The seminiferous epithelium contains both germ cells and somatic Sertoli cells, which (in vivo) create a network of tight junctions and form the blood-testis barrier. In the Ogawa device, this barrier was simulated by a porous membrane through which the seminiferous tubules were supplied nutrients. In the device, the testis tissue maintained spermatogenesis for 6 months, kept producing testosterone, and responded to stimulation by luteinizing hormone. In addition, the produced sperm were able to generate healthy mouse offspring. This work demonstrates that a simple microfluidic device can help maintain normal physiologic conditions of intact tissues for a long time. The Le Gac lab built a testis-on-a-chip model using functionally regressed adult human testicular tissue from gender dysphoria patients^100^. Microfluidic cultures of multi-tubular tissues (MTT) and single seminiferous tubules were kept for several days. They assessed the viability of MTTs over 11 days and characterized the porosity, toxicity, and retention capacity of the porous barrier design. A team led by Woodruff and Borenstein developed a modular organ-on-a-chip platform that allowed them to model the human female’s reproductive tract and 28-day menstrual cycle^101^. Flow was controlled using pneumatic valves and electromagnetically-actuated micropumps. The platform could be used to study a single tissue (e.g. ovarian explants from mice), or multiple (up to 5) tissues to simulate a hormonally-coupled ex vivo female reproductive tract (e.g. murine ovary human fallopian tube, endometrium, ectocervix, and liver tissues) for 28 days. Their data suggested that the integration of multiple tissues changed the ovarian hormone expression in the upstream ovarian tissue and/or that downstream reproductive tissues (fallopian, endometrium, and/or ectocervix) consumed ovarian hormone secretions.

Many researchers have used microfluidic devices to isolate, culture, stimulate, and analyze pancreatic islets. The pancreatic islets (or islets of Langerhans) are ~100–200-µm-diam. regions of the pancreas that contain ~1000–10,000 hormone-producing cells and maintain the glucose levels in the body. Multiple different hormones (glucagon, insulin, and somatostatin, among others) are secreted by at least five different cell sub-types that are spatially arranged in specific patterns. Importantly, the islets can be removed from the pancreas and studied in vitro. Kennedy and colleagues developed the first microfluidic chip for online, automated monitoring of insulin secretion from single islets based on a capillary electrophoresis immunoassay for insulin downstream of the islet culture chamber^102,103^. Their glass-based chip was later parallelized to accept up to 15 individual islets and independent assays^104^. The Piston lab designed a PDMS microfluidic device featuring a pneumatic actuator that was able to trap and hold a single pancreatic islet between two separate fluid streams, allowing for the application of asymmetrical stimuli to the islets (Fig. 4c–f)^105^. Glucose stimulation applied to one side of the pancreatic islet revealed coordination of intracellular Ca^+2^ activity oscillations limited to that side. Using a droplet-microfluidics setup, this group also sampled zinc secretions from single pancreatic islets by low flow rates and by fluid collection of aqueous-in-oil droplets formed downstream in a way that minimized dilution of secreted molecules^106^. Ismagilov and co-workers developed the “chemistrode”, a plug-based microfluidic device that enables stimulation, recording, and analysis of cell culture surfaces with high spatial and temporal resolution^107^. They used the device to perform measurements of insulin secretion from single murine pancreatic islets at 0.67 Hz (Fig. 4g, h). Eddington, Wang and co-workers designed a microfluidic chamber containing an array of 500-µm-diam. microwells that traps small groups of islets in each well (a total of 25 islets/chamber)^108^. With the device, they obtained insulin secretion profiles from the ensemble of all the islets in the chamber and optically recorded Ca^+2^ oscillations from individual islets. This team later implemented more efficient hydrodynamic trapping schemes to improve the islet imaging and screening inside the device^109,110^. Rocheleau’s group found that, by culturing the pancreatic islets under flow in a microfluidic chamber, the flow penetrating the islets prevented the deterioration of the endothelial vascular structures within the islets^111^. Easley’s group demonstrated a passively-operated PDMS microfluidic device for sampling hormone secretions from eight individual murine pancreatic islets in parallel^112–114^. Islet volume measurements with confocal reflectance microscopy revealed that insulin secretion only had limited correlation to islet volume, suggesting that paracrine signaling and/or the tissue microenvironment within the islet play a more significant role^112^.

Adipose tissue is not simply a passive reservoir for energy storage, but also a complex and highly active metabolic and endocrine organ – containing adipocytes, connective matrix, nerve tissue, stromovascular cells, and immune cells – that is a key player in metabolic disorders such as Type 2 Diabetes Mellitus (T2DM)^115^. By mimicking the adipose tissue microenvironment, microfluidics could help elucidate some of these cellular and biomolecular interactions in both the normal and the disease state^116,117^. The Easley lab fabricated an 8-channel PDMS-molded device^113^ and a PDMS valve multiplexer^114^ to demonstrate the automated delivery of treatments and sampling of secretions from adipose tissue explants (a mimic of the circulation in the endocrine system). A team led by Quake and Elvassore demonstrated a microfluidic platform featuring microvalves and eight independent tissue culture chambers for the detection of glucose uptake from human adipose tissue biopsies^118^ (Fig. 4i, j). They stimulated adipose tissue samples (healthy compared to a T2DM patient) with overlapped steps of both 100 nM insulin and glucose. They showed a significant decrease in outlet glucose concentration from the insulin-stimulated chamber with respect to the non-stimulated one in normal tissue, and not in the tissue from the diabetic patient (Fig. 4k).

### Intestinal tissue

The gut’s complex architecture (it is formed of four concentric layers, the innermost of which is the mucosa containing villi or folds as well as glands) hosts ~4000 strains of microbes that play diverse roles in immunity and metabolism. Several labs have attempted to build “gut-on-a-chip” systems starting from cell lines or stem cells (see review of gut and microbiota on a chip^119^), but these systems often lack critical biomolecular interactions with ECM, bacteria, and immune cells. A team led by Greenman and Jacobsen designed a “dual flow” PDMS device to independently perfuse the luminal (innermost) and serosal (external) sides of full-thickness human intestinal tissue for up to 72 h^120^. Physiologically relevant maintenance of viability, cell proliferation, and calproctin levels (a measure of the inflammatory state) was demonstrated throughout the experiment. Such intact-tissue chip platforms could potentially help address human gut disorders and their therapeutic interventions in ways that cannot be addressed by current models based on human stem cells or animal cells.

### Skin and hair on a chip

Most tissue models of the skin are based on static culture, which limits their applications in toxicity and compound screening. The application of microfluidic devices to intact skin tissue may provide a more relevant system. Skin is a highly stratified organ that covers the outer surface of animals and consists of several cell types besides keratinocytes – including endothelial cells (forming blood capillaries), sensory nerve cells, muscle cells, immune cells, and resident bacteria – as well as secretory glands and follicular (hair-growing) structures. Lindner’s group used a microfluidic device with a built-in micropump to dynamically perfuse human foreskin biopsies as well as biopsies of single hair follicular units^121^. The researchers observed an abundance of proliferative cells after 14 days of culture and indicated that dynamic perfusion of an intact tissue model prevented tissue disintegration.

### Plant biology

**Fig. 5:**
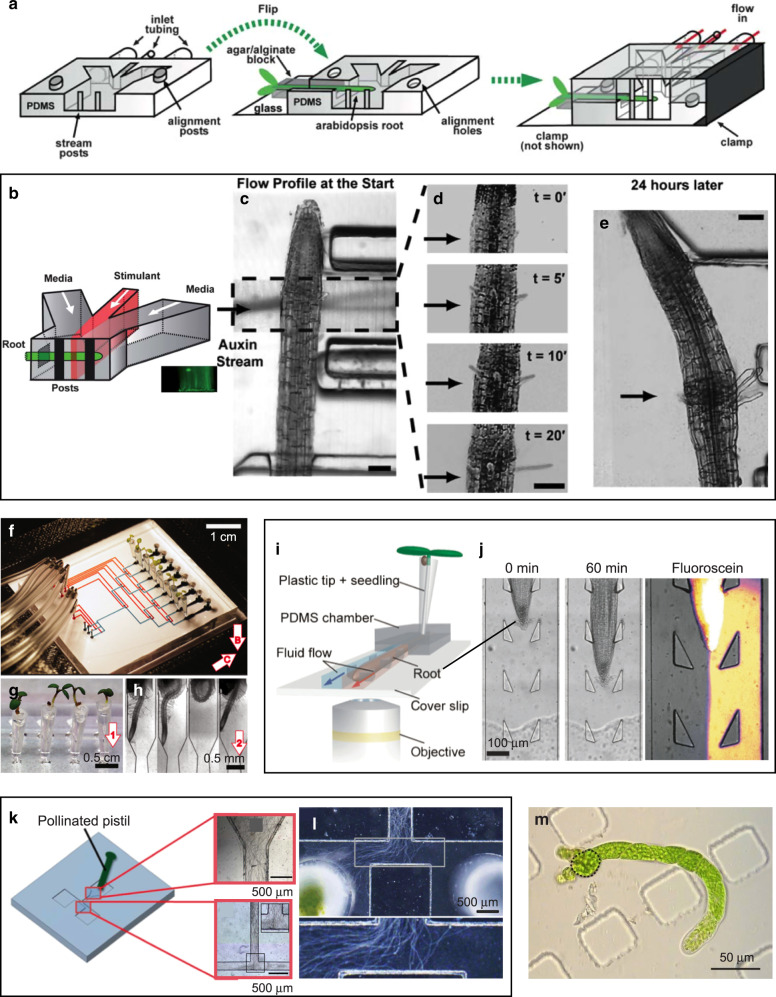
Microfluidic devices for probing plant tissues. **a**–**e** A microfluidic device for interrogating *Arabidopsis* roots. **a** Schematic of the assembly of the microfluidic device around a live *Arabidopsis* root using two PDMS molds (one forming the bottom half and the other forming the top half of the device). The root channel was filled with agar/alginate. Both PDMS molds were attached to glass slides (glass slide for top mold is not shown). **b** Schematic of the microfluidic platform for a chemical stimulation experiment with laminar flow (stimulant shown in red). **c**–**e** Local hair growth enhancement by stimulation with a 10–20-µm segment of auxin close to the tip of an *Arabidopsis thaliana* root at the start of the experiment. **c** Start of the experiment. **d** Zoomed-in region of auxin stimulation showing time-lapse of hair growth after 0, 5, 10, and 20 min. **e** Micrograph of the root taken 24 h after the auxin stimulation. Black arrows denote the position of the auxin laminar stream. Adapted with permission from ref. ^124^. **f**–**h** The “Root Chip”. **f** PDMS chip with eight mounted live plants. For illustration purposes, pneumatic, and flow channels are filled with red and blue dyes, respectively. **g** Close-up of plants in conical cylinders filled with agar. Arrow indicates the growth direction of the root. **h** Closeup of microchannels containing roots at 7 days after germination. Adapted with permission from ref. ^125^. **i**, **j** A Root Chip with heterogeneous laminar flow. **i** 3D schematic of the device illustrating the simultaneous delivery of two different reagents to either side of a growing root by two fluid streams (red and blue) within the microchannel. **c** Time-series illustrating growth and guidance of an Arabidopsis root through the array of flexible pillars (0 and 60 min) and visualization of heterogeneous laminar flow using water and fluorescein. Adapted with permission from ref. ^129^. **k**, **l** A microfluidic device for studying chemoattraction in pollen tubes. **k** Schematic of the microfluidic device. A pollinated pistil is placed in the inlet such that pollen tubes can emerge from the cut end of the style and enter the flow channel (inset images). **l** When an ovary was placed in the left reservoir, pollen tube growth oriented towards it. Adapted with permission from ref. ^130^. **m** Moss protoplast regeneration in PDMS chambers. A plant regenerated from a protoplast rebuilt its cell wall and underwent several cell divisions within 9 days. The initial protoplast is outlined in a dashed black line. Figure courtesy of M. Benzanilla and S.-Z. Wu

With microfluidics, plant biologists have created “soil-on-a-chip” platforms that simulate water and solutes uptake as well as chemical sensation and other interactions by plants^122,123^. Ismagilov and colleagues built a two-part microfluidic device in which the plant roots are inserted into a microchannel^124^. The microchannel features three inlets that permit local perfusion of the root with a solution of choice using heterogeneous laminar flow (Fig. 5a, b). They demonstrated local exposure of live roots of *Arabidopsis thaliana* (a model organism in plant biology and genetics, the first plant genome to be sequenced, and a suitable tissue for light/fluorescence microscopy because the seedling and the roots are translucent). Local exposure to auxin (Fig. 5c) resulted in local GFP expression in the root and local epidermal hair growth (Fig. 5d, e). Quake and Meier led a team that fabricated the “RootChip”, a multiplexed version of Ismagilov’s device that featured integrated PDMS valves to route the stimuli and inputs for eight Arabidopsis roots simultaneously^125^ (Fig. 5f–h). Perfusion with glucose led to alterations in intracellular sugar levels mainly in the root tip in response. Benfey’s group demonstrated a microfluidic/computational platform for up to 64 *Arabidopsis* seedlings that permits automated, high-throughput imaging of gene expression of their roots over several days^126^.

Subsequent microfluidic platforms and studies have helped obtain mechanistic insights into cell–cell signaling in response to local environmental stimuli, such as nutrients or herbicides. Parashar and Pandey^127^ designed a microfluidic device for co-cultivation of *Arabidopsis* roots and plant pathogens, such as the oomycete *Phytophthora sojae*, in order to probe their interactions. Similarly, Aharoni’s team used a microfluidic device with nine chambers to image fluorescently labeled bacteria (*Bacillus subtilis*) as they colonized the root of *Arabidopsis* within the device in real time^128^. The Grossman group devised a variation of the Root Chip design that guided the growth of the roots in a straight line by means of microposts and allowed for heterogeneous laminar flow into the root channel, enabling one half of the root to be exposed to one stimulus and the other half of the root to another stimulus^129^ (Fig. 5i, j). Kaji and colleagues^130^ fabricated a T-shaped microchannel in PDMS to probe the growth of pollen tubes in response to chemoattractants (Fig. 5k); placement of an ovary in one of the chambers leading to the “T” produced striking directional growth of the pollen tubes towards the ovary (Fig. 5l). Geitmann’s group designed PDMS microchannels with narrowings that constricted the tips of growing pollen tubes; based on the deformation of the gaps, the force exerted by the elongating tubes was determined using finite element modeling^131,132^. Bezanilla and co-workers successfully grew and characterized the growth rates of three different types of moss (chloronemata, caulonemata, and rhizoids) inside PDMS microfluidic devices^133^ (Fig. 5m). Similarly, a team led by Lee and Nicolau used microfluidic mazes to simulate the growth of fungi through soil^134–136^. Aebi, deMello, and co-workers probed bacterial-fungal interactions and fungicidal activity of *Bacilus subtilis* at the single-cell level with a PDMS microfluidic device^137^.

## In vivo studies

### Small-animal research

**Fig. 6:**
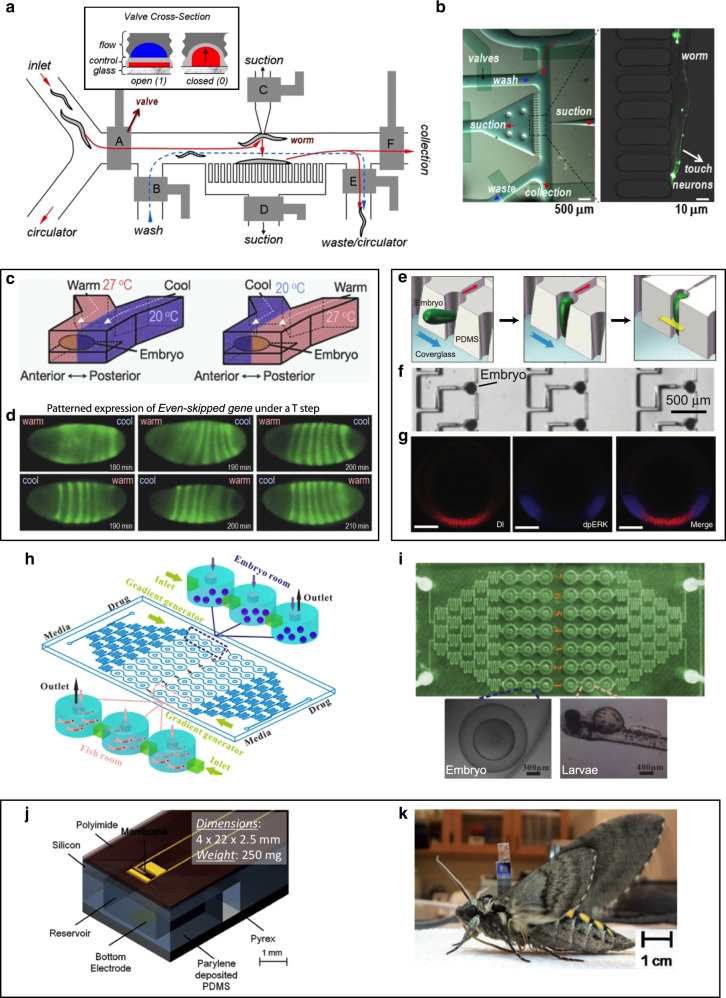
Microfluidic devices for small-animal research. **a**, **b** Microfluidic worm sorter. **a** Microfluidic worm-sorter schematics. The sorter consists of control channels and valves (gray) that direct the flow of worms in the flow channels in different directions with valves (labeled **A**–**F**) in order to isolate and capture individual worms, image them, and finally sort to waste or collection. **b** Micrograph of the device during image acquisition from a trapped worm. Adapted with permission from ref. ^147^. **c**, **d** PDMS microfluidic device for *D. melanogaster* embryo development in response to a temperature step. **c** Schematic of the experimental setup. **d** Fluorescence micrographs of *D. melanogaster* gene expression after exposure to a temperature step that was applied before the first image. Remarkably, over time the embryos exposed to inverse steps ended up expressing the same (correct) patterns. Adapted with permission from ref. ^153^. **e**–**g** A microfluidic array for large-scale trapping of *Drosophila* embryos. **e** Schematic showing the embryo trapping process: the flow (blue arrows) guides the embryo into the trap and orients it vertically; finally, the trap contracts and secures the embryo. **f** Micrograph showing a section of the array with trapped embryos. **g** Confocal images of signal transduction and morphogen gradients in dorsoventral patterning activated by Dorsal (Dl, an NF-κB transcription factor, which subdivides the embryo into three germ layers) and phospho-MAPK (dpERK). Adapted with permission from ref. ^154^. **h**, **i** A microfluidic platform for real-time monitoring of drug-induced developmental toxicity in zebrafish. **h** Schematics of the zebrafish platform, which includes two independent zones, each with a media inlet, a drug inlet, a gradient generator and seven series of fish tanks (each concentration with three tanks). **i** Photo of the microfluidic chip (top) and micrographs (bottom) of an embryo (left) and larvae (right) in the chip. Adapted with permission from ref. ^156^. **j**, **k** Microfluidics for engineering insect flight metabolics, implanted at an immature stage. **j** Schematic of the electroactive microwell drug delivery system. **k***M. sexta* moth successfully emerged from the pupa with an implanted device. Adapted with permission from ref. ^164^

Some multicellular model organisms are small enough that they can be manipulated at a submillimeter scale within microfluidic channels (see review of microfluidics for small, multicellular model organisms^138^). In this approach, the microfluidic platform keeps the animal alive, while the animal itself maintains tissue homeostasis. The roundworm *C. elegans* – a ~1-mm-long transparent nematode widely used as a model invertebrate organism – can be manipulated very efficiently with microfluidics (see reviews of microfluidics for *C. elegans*^139–143^). The Bargmann lab pioneered the use of microfluidics in combination with *C. elegans* with an oxygen gradient generator that triggered different worm behaviors depending on the oxygen concentration^144^. The group also characterized the various locomotive behaviors of *C. elegans* under a wide range of spatiotemporal chemical stimuli generated within a microfluidic chamber^145^. Channels have been designed that trap the worms unidirectionally; the worm enters a narrowing channel until it gets stuck, leaving the head/tip accessible to various fluids, and the exposure to fluids can be switched by changing the pressures of a heterogeneous laminar flow^146^. Many labs have used PDMS microactuator technology to immobilize or exert forces on the worms at high throughput (a pneumatically actuated PDMS membrane pushes the worms down), as well as to switch fluid application with PDMS valves. Worm-screening devices have been developed that isolate, immobilize, image (for phenotyping), and/or perform femtosecond laser microsurgery (e.g. axotomy), as well as sort worms into multiwell plates (e.g. for drug screening) – all within a fraction of a second per worm, an improvement of several orders of magnitude over previous manual procedures^147–149^ (Fig. 6a, b). A different design based on the encapsulation of the worms in droplets also achieved very high processing throughputs^150,151^ but did not allow for the focal application of drugs or of mechanical stimuli. A microfluidic device containing a post array was used to simulate the locomotive behavior of nematodes in dirt^152^.

In the embryo during development (initially, formed of just a few cells), the microenvironment of every cell constantly changes following a finely orchestrated gene expression program that cannot be accurately reproduced in traditional Petri dishes. On the other hand, microfluidic systems – with their ability to deliver fluids to particular locations on-demand – offer an enormous potential to investigate development in vitro. Embryogenesis in *Drosophila melanogaster* (the common fruit fly) has been extensively studied due to its small size and short generation time. Ismagilov and colleagues used spatial patterns of gene expression in a *Drosophila* embryo to study perturbations in biochemical networks in a microfluidic device^153^. A *Drosophila* embryo was placed inside a microfluidic channel that had two inputs, one for a warm (27 °C) solution and the other for a cool (20 °C) solution that slows development. The embryo was exposed to the two streams simultaneously, producing a non-physiological temperature “step” that caused different parts of the embryo to develop at different rates (Fig. 6c). Some embryos were exposed to warm solution in the anterior part, and others in the inverse orientation. Although, initially, the gene expression patterns in the anterior and posterior parts were rather different, after ~200 min the patterns were very similar regardless of the environmental differences that the embryos had been exposed to (Fig. 6d). Lu’s group has developed a hydrodynamic trapping approach for large-scale ordering and orientation of *Drosophila* embryos^154^; they could fill ~90% of the traps with embryos (impressively, in the same orientation) of a 700-trap array and visualize fluorescent expression patterns for various genes while the embryos were in the traps (Fig. 6e–g). In addition, various microfluidic designs have also been presented that detect, recognize, sort, and analyze multicellular plankton, although most of the work has been on unicellular plankton (see review on microfluidics for plankton^155^). A number of groups have applied similar concepts to the trapping and toxicology testing of embryos of zebrafish^156–162^, a vertebrate model (see review on microfluidics for zebrafish^163^) (Fig. 6h, i).

The large flying moth *Manduca sexta* is a widely used model for the neuroartificial control of flight using microsystems because big loads do not significantly affect flight patterns (~1 g is about half its body mass). In 2008, Erickson’s group implanted a microfluidic device into the thorax of *Manduca sexta* pupae that delivered reversibly paralyzing agents to the adult-stage insects^164^ (Fig. 6j, k). The devices measured 4 mm × 22 mm × 2.5 mm and weighed 250 mg (Fig. 6j). They delivered agents such as l-glutamic acid (LGA), l-aspartate acid (LAA), or γ-aminobutyric acid (GABA) that serve as excitatory neurotransmitters at insect skeletal neuromuscular junctions, but at the high concentrations, they act as venoms, as when produced by many spiders and wasps. The device was implanted at the pupal stage because the wound heals better – and presumably integrates better with the physiology of the animal – at this developmental stage than at the adult stage. Once the moth reached the adult stage (Fig. 6k), its respiratory CO_2_ output was monitored in a chamber where it was allowed to flap its wings (i.e. “fly” without moving). The neurotoxins (LGA, LLA or GABA) were loaded in a reservoir capped by a gold membrane. The membrane could be dissolved electrochemically on command by application of a small voltage, which caused the release of the chemicals into the thorax of the insect and (reversible) wing paralysis within 90 seconds.

### Microfluidic skin sensors

The rapid development of wearable technologies for physiological monitoring is transforming consumer wearable devices into clinical-grade tools that offer deep insight into overall health status and physical performance. Body-worn microfluidic sensors represent an emerging class of devices that enable sampling and analysis of biochemical species found in noninvasive biofluids such as sweat, tears, and saliva. Sweat, in particular, offers perhaps the most promising non-invasive window into the human physiological health state^165^. Sweat from the eccrine sweat glands (widely distributed across the human body) contains a rich composition of biomolecular targets (e.g., electrolytes, metabolites, hormones, and drugs/toxins), which vary in concentration and composition in response to changes in health or physical activity^166^. Skin-interfaced microfluidic sensors utilize electrochemical sensors or colorimetric assays to detect and quantify sweat biomarkers of interest. Prior to the emergence of such platforms, sweat analysis relied upon absorbent pads and external laboratory equipment for biomolecular quantification. By integrating miniaturized, flexible electronics and wireless communications capabilities with these sensors, these skin-interfaced microfluidic platforms can perform real-time, multimodal analysis of physiological signals at the point-of-use.

**Fig. 7:**
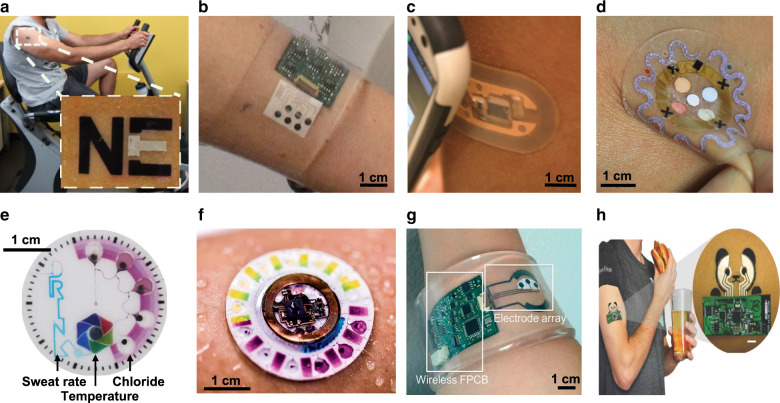
Skin-interfaced microfluidic sensors. *Form factors*. **a** Epidermal temporary-tattoo electrochemical sensor for monitoring sweat lactate concentrations. Adapted with permission from ref. ^167^. **b** Wrist-mounted platform with integrated electrochemical sensors and flexible electronics for quantification of glucose, lactate, potassium, and sodium concentrations in sweat. Adapted with permission from ref. ^170^. **c** An adhesive bandage-style wearable sensor for wireless time-sequenced monitoring of sweat chloride levels. Adapted with permission from ref. ^171^. **d** Stretchable, epidermal microfluidic device conformally interfaced with the skin with integrated colorimetric assays for quantifying sweat chloride, lactate, pH, and glucose as well as sweat rate and total sweat loss. Adapted with permission from ref. ^172^. *Advanced Capabilities*. Examples of epidermal microfluidic devices with **e** passive capillary burst valves for time-sequenced analysis of sweat and **f** NFC-powered wireless electrochemical sensors for multiplexed sweat analysis. Adapted with permission from refs. ^166,177^. Integration of pharmacological sweat stimulation by iontophoresis enables continuous, wireless analysis of **g** chloride or **h** alcohol and glucose in sweat. Adapted with permission from refs. ^180,181^

Early skin-interfaced biosensors directly interfaced electrochemical sensors to the epidermis. Wang’s group fabricated screen-printed electrodes on thin adhesive substrates, similar to a temporary tattoo, to facilitate a conformal skin contact supported by a band-based wireless transceiver (Fig. 7a)^167,168^. Other groups harnessed emerging advances in flexible electronics to develop fully integrated electrochemical sensing platforms in wristband- or bandage-based form factors^169^. The Javey group’s pioneering work on a ‘smart wristband’ integrated a flexible printed circuit board (flexPCB) with electrochemical sensors mounted on a flexible polyethylene terephthalate (PET) substrate to monitor sweat glucose, lactate, sodium, and potassium (and temperature) (Fig. 7b)^170^. The thin construct of the PET substrate, supported by the external pressure from the band, enabled sustained direct contact between the sensor and skin during exercise. Heikenfeld’s group developed a prototypical Band-Aid style platform to monitor sweat chloride concentration via a wirelessly powered (radio-frequency identification, RFID) electrochemical sensor (Fig. 7c)^171^. Here, sweat could wick from the skin to the sensor via a porous adhesive layer, which, in turn, maintained conformal contact with the epidermis. These platforms offered versatile episodic and/or continuous monitoring of exercise-induced sweat bioanalyte variations.

The time-dynamic variation of sweat constituents necessitates real-time monitoring; however, continuous analysis requires constant transport of newly generated sweat across a sensor surface. The absence of an efficient sweat transport mechanism limited the reliability and accuracy of these early platforms. Integration of established lab-on-chip designs with soft, elastomeric materials (e.g. silicones) enabled the emergence of sophisticated microfluidic sampling platforms. Characterized by skin-like material properties, these devices form an intimate, non-irritating interface with the skin. This, in turn, facilitates the direct collection of sweat via a water-tight seal, while also supporting seamless wear and robust device operation under a variety of conditions (e.g., clinical, athletic, home). First developed by the Rogers group, these epidermal microfluidic systems (‘epifluidic’ systems) passively collect, transport, and store microliter quantities of sweat (Fig. 7d)^172^. Networks of microfluidic channels, sensing reservoirs^166^, and colorimetric^173^ assays enable the concurrent measurement of sweat constituents as well as local sweat rate and instantaneous sweat loss (Fig. 7e). Refinements incorporating pressure-based valves^174,175^, stretchable electronics, and near-field communications (NFC) capabilities^176^ with simultaneous colorimetric and electrochemical analysis^177,178^ expanded the accuracy of multimodal sensing in a form-factor suitable for real-time, extended-wear applications (Fig. 7f). As described in complementary reviews^166,169,179^, continued developments of epidermal microfluidic devices have expanded the range of detectable sweat biomarkers and physiological parameters.

These representative skin-interfaced microfluidic devices depict the key technologies in an active area of academic and commercial research. Continued progress requires attention in addressing long-term sensor accuracy, correlating time-dependent concentration variations with physiological relevance, and reliable operation across the broad application space. Indeed, a key limitation is the reliance upon active sweat stimulation by either physical exertion, elevated temperatures, or chemical inducements^169^. Recent work demonstrates the utility of pharmacological sweat stimulation for continuous monitoring in clinical (chloride^180^, Fig. 7g) and consumer (glucose and alcohol^181^, Fig. 7h) applications; however, alternative sampling routes are still necessary to enable broad deployment for active and sedentary applications. Such fully-integrated, wearable microfluidic sensors show wide-ranging potential in expanding clinical diagnostic capabilities, improving personal wellness, and enhancing athletic performance monitoring.

### Microscale drug delivery to live tissues in vivo

**Fig. 8:**
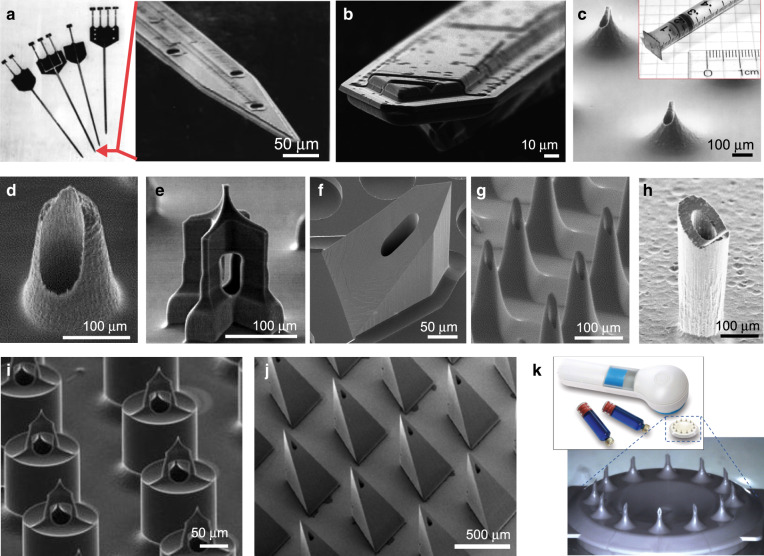
Hollow microneedles for microfluidic drug delivery. **a** The first hollow microneedles were developed in silicon for in vivo neural recordings. Adapted with permission from ref. ^185^. **b** The first transdermal hollow microneedles, also developed in silicon. Adapted with permission from ref. ^193^. **c**–**i** Various hollow silicon microneedles. **c** Adapted with permission from ref. ^194^. **d** Adapted with permission from ref. ^196^. **e** Adapted with permission from ref. ^198^. **f** Figure contributed by J.G.E. Gardeniers and A. van der Berg. **g** Adapted with permission from ref. ^200^. **h** Adapted with permission from ref. ^201^. **i** Adapted with permission from ref. ^202^. **j** PMMA hollow microneedles fabricated by the LIGA process. Adapted with permission from ref. ^203^. **k** Polymer hollow microneedles by 3M. The material and fabrication process are proprietary. Adapted with permission from ref. ^205^

The small size and precise fluid control of microfluidics has made this technology an extremely fertile paradigm for drug delivery research (see review of microfluidic drug delivery^182^). Developed for various applications since the 1990s, the earliest implementations were microfabricated needles (“microneedles”), in various shapes, forms and materials, from solid to hollow, and from metal to polymeric and even dissolvable (see review of microneedles for drug and vaccine delivery^183^). Hollow microneedles essentially constitute microfluidic devices that directly inject drugs into tissue^183,184^. In 1997, Wise and colleagues microfabricated an in vivo multichannel silicon probe for neural tissue that could inject kainic acid and GABA while simultaneously recording electrical signals from neurons and electrically stimulating neurons^185^ (Fig. 8a). Fürjes’ group fabricated a similar silicon fluidic probe with integrated platinum microelectrodes^186^; they locally injected bicuculline in the cortex and in the thalamical regions of rat brain in vivo, while simultaneously recording the electrical signals of the stimulated neurons on four different electrical channels. Shain and co-workers demonstrated the infusion of a cell marker cocktail at constant pressure into rat neocortex using microfluidic silicon probes^187^. These early neural probes suffered from foreign-body rejection but helped lead to the understanding, and eventually resolution of, the associated biocompatibility issues. Xu’s group successfully tested parylene-coated 3D neural silicon probes with integrated microchannels in rats^188^. Isaacson and colleagues proposed early on the integration of microchannels into silicon-based cortical probes in order to alter the biochemical composition of tissue around implanted devices^189^, with the goal of controlling the reactive astroglia response, e.g. by delivery of dexamethasone^190^. Patil’s group placed a 16-microelectrode silicon probe inside a fused-silica catheter (outer diam. 165 µm, inner diam. 127 µm) that served for fluid delivery in addition to recordings^191^. A team led by Zengerle integrated a micropump with neural silicon microprobes for drug delivery in small animals^192^.

Microfabricated needles (“microneedles”) have found wide applicability in the field of transdermal drug delivery. The primary barrier to transport across the skin is the stratum corneum, the outer 10–15-µm-thick layer of the skin formed of dead keratinocytes. Microneedles for transdermal use have precious advantages with respect to traditional hypodermic needles: (1) they reduce tissue damage; (2) they reduce insertion pain and eliminate bleeding (nerves and blood vessels are only found in deeper tissue); (3) they are more likely to be accepted by people who have phobia of needles or have concerns about them, such as a newborn’s parents or people who have just come into contact with modern medicine; (4) they can be integrated with microfluidic devices or storage microchambers (if they are hollow); (5) they can be multiplexed, i.e. multiple applications at once or at multiple spots very close together, so that large doses can be administered as the sum of many small doses; and (6) they can be incorporated in a user-friendly “patch” format for self-administration, which would allow non-experts – even people in remote villages and the elderly – to vaccinate themselves.

The first researchers in this area had to solve various technical challenges before they could address the biomedical problems for which the microneedles were intended. Lin and Pisano^193^ demonstrated the first (silicon) microneedles intended for transdermal use (Fig. 8b). Soon afterward, Liepmann’s group integrated micropumps with Pisano’s microneedles^194^ and achieved continuous outflow pumping at 2 nL/s for over 18 h of 15,000 cycles. However, because the microneedles were thin and made of silicon, they were limited to very shallow channels and to in-plane, brittle microneedles that were difficult to connect to tubing. In 2003, Allen, Prausnitz, and co-workers reported the first out-of-plane hollow microneedles (made in metal and featuring a central aperture), which could be used to deliver arbitrary solutions and be connected through the back of the wafer without complicated interfaces^195^. Figure 8c shows an example of out-of-plane, 200-µm-tall hollow microneedles fabricated in silicon by Liepmann’s group^194^. The 40-µm-diam. hole at the center of the tips is a through-hole, so the tips can be fed from the opposite side of the wafer from a regular syringe (see inset in Fig. 8c) to inject compounds past the stratum corneum. Morrissey and colleagues produced silicon microneedle arrays by deep-reactive ion etch (DRIE)^196^ (Fig. 8d); the microfluidic channels were created in a separate wafer, also by DRIE, that was bonded to the microneedle array’s backside (needle height 320 µm, width of needle hole ~50 µm). Yang and colleagues were able to integrate a piezoelectric insulin pump with a silicon microneedle array^197^. However, these vertical hollow microneedle designs tended to get clogged by the tissue during insertion, which changed the fluidic resistance of the array (since they were all connected to the same inlet). Stemme’s group developed a creative hollow 210-µm-high microneedle design whereby the tip’s hole was protected from clogging by a sharp micro-hat that also aids in tissue penetration^198^ (Fig. 8e). To dodge the clogging issue, several other groups presented various designs with ingenious shapes. Van der Berg’s group reported an imaginative etching process for fabricating 350-µm-high out-of-plane silicon microneedles with 70-µm-wide elliptical side-openings^199^ (Fig. 8f). Smith, Isseroff and co-workers achieved silicon microneedles with a hypodermic needle-like aspect by etching silicon on a Pyrex wafer and shifting the bore hole^200^ (needle height 200 µm, width of needle hole 10 µm) (Fig. 8g). Cabodevila’s group was able to create beveled microneedles^201^ (Fig. 8h). The Jullien lab developed a variation of Stemme’s design^202^ (Fig. 8i). Moon, Lee and colleagues developed an array of tetrahedron PMMA microneedles using LIGA (X-ray lithography of PMMA) in two successive exposures, one orthogonal and one inclined exposure^203,204^; PMMA increased the biocompatibility of the microneedles and minimized the risk of breakage (Fig. 8j). As shown in the inset, these microneedles are deep enough to penetrate through the stratum corneum, the top skin layer of keratinized dead cells, and reach the blood vessels for potential blood extraction (shallower microneedles for diffusive drug delivery are also possible).

Some transdermal microneedles with a loading device are now commercially available. For example, the 3M system depicted in Fig. 8k features 12 polymer microneedles that are 1500 µm in height and that can transdermally deliver up to 2 mL of a protein solution (20 cp of viscosity) in 2–4 min or up to 0.9 mL of a 80 cp solution in 1–2 min^205^. A team led by Prausnitz has recently developed a novel strategy that locally triggers “effervescent” chemical reactions in order to release biodegradable (solid) microneedles, an approach that they showed provides women with better access to contraception^206^. The patch incorporates an effervescent backing that causes rapid separation of microneedles from the patch, enabling separation within 1 min. The microneedles are made of poly(lactic-co-glycolic) acid, a biodegradable polymer that slowly releases the contraceptive hormone levonorgestrel for ~1 month. Importantly, 90% of women (*n* = 10) preferred the microneedles to the oral contraceptive pill.

**Fig. 9:**
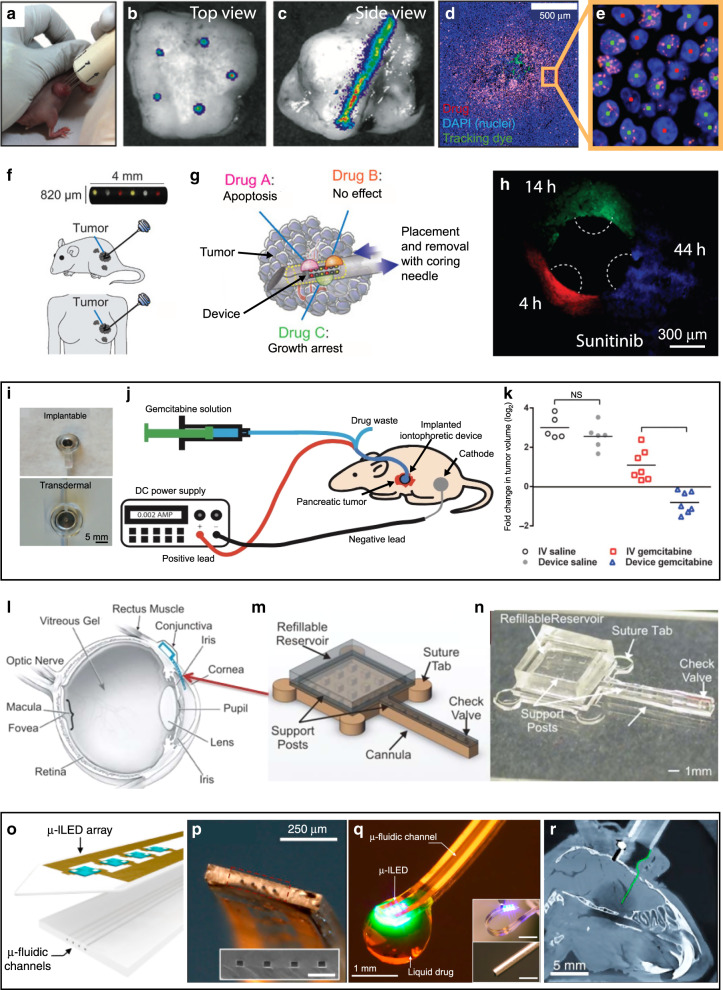
Microfluidic in vivo drug delivery. **a**–**e** The CIVO device developed with PresageBio. **a** The CIVO device, which consists of an array of hypodermic needles, being used for injection to a mouse tumor. **b** Top-view of a tumor site after injection of microliter-quantities of the drug; the green label is injection tracking dye added to the drug solutions. **c** IVIS imaging of the intact tumor showing the column-like distribution of the tracking dye signal from a single needle spanning the *z*-axis of the tumor. **d** The blue label is nuclei labeled with DAPI; the green label is an injection tracking dye; the orange label is a drug-specific biomarker. **e** The resulting images were processed by a custom image analysis platform, which classifies the cells within each region of interest as biomarker-positive (green dots) or biomarker-negative (red dots). Adapted with permission from ref. ^71^. **f**–**h** An implantable microdevice to perform multiplexed in vivo drug sensitivity testing in tumors. **f** A device with multiple (up to 16) reservoirs that can be implanted into a tumor using a coring needle. **g** Each reservoir is loaded with microdoses of anti-cancer agents, which are diffusively released upon implantation. **h** Release of a microdose of sunitinib (50% in PEG-1450) into BT474 tumors at three time points. Adapted with permission from ref. ^70^. **i**–**k** Local iontophoretic administration of cytotoxic therapies to solid tumors. **i** Photographs of the microfluidic iontophoretic devices used to administer drugs by internally after implantation (top) or transdermally (bottom). **j** Experimental setup for delivery of gemcitabine to pancreatic tumors. **k** Graph depicting the fold change in tumor volume as a measure of the efficacy after treatment of pancreatic cancer PDX mice with gemcitabine, I.V. gemcitabine, device saline, or I.V. saline twice per week for 7 weeks. ***p < 0.0001. Adapted with permission from ref. ^207^. **l**–**n** Treatment of ocular diseases with a refillable microfabricated drug delivery device implanted under the conjunctiva. **l** Illustration of device placement on the temporal side of the eye, providing easier access for a refill and improving patient comfort. **m** 3D schematic of the device. **n** Photograph of the device. Adapted with permission from ref. ^208^. **o**–**r** A wireless optofluidic system for programmable in vivo pharmacology and optogenetics. **o** Schematic diagram of the integration of a soft microfluidic probe with a flexible array of µ-ILEDs (inorganic LEDs, each with lateral dimensions of 100 µm × 100 µm and thicknesses of 6.54 µm) and metal interconnect traces on a 6-µm-thick film of PET. **p** The tip end of an optofluidic probe. (Inset) SEM of the outlets of the microfluidic channels. **q** Optofluidic neural probe during simultaneous drug delivery and photostimulation. (Insets) Comparison of this device (top) with a conventional metal cannula. **r** Computed tomographic x-ray images of the mouse model with an optofluidic neural probe (colorized green) implanted into the brain. Adapted with permission from ref. ^214^

The short length of microfabricated needles can be a limitation for intratumoral injection. Olson and colleagues have developed a microinjection technology (termed CIVO) that enables the simultaneous assessment of up to eight drugs or drug combinations within a single solid tumor in vivo^71^. CIVO is a device that features an array of hypodermic needles (Fig. 9a), which enables the introduction of microliter-volumes of multiple drugs transcutaneously into discrete locations within a growing tumor in a living subject (Fig. 9b). Figure 9c shows the column-like distribution of the tracking dye signal from a single needle spanning the *z*-axis of the tumor. The tumor response to a microinjected drug at an injection site is shown in Fig. 9d, e.

The CIVO technology requires one needle per drug, which can be limiting for small tumors or difficult-to-access tumors. The Langer group has developed a multi-reservoir (up to 16) device that can be lodged inside a coring needle and implanted into the tumor. There, the reservoirs release microdoses of single agents or drug combinations^70^ (Fig. 9f, g). Thus, the device can perform drug sensitivity testing of several anticancer agents simultaneously inside the living tumor while still inside the host. To optimize release, the drugs were mixed with polymers. Figure 9h demonstrates the release at three time points of a microdose of sunitinib (a fluorescent cancer drug) into BT474 tumors using a formulation of 50% PEG-1450. Such devices could help identify optimal drug therapies for individual patients prior to systemic treatment and could also be used in clinical drug development to more rapidly obtain efficacy data on new compounds.

Chemotherapy for cancer patients typically consists of the systemic administration of cytotoxic compounds that results in toxicity to off-target organs and poor tumor perfusion. In order to improve the efficacy of cytotoxic therapies and to mitigate the toxic side effects, a team led by De Simone developed a microfluidic device for the localized iontophoretic delivery of chemotherapeutic agents^207^ (Fig. 9i–k). The iontophoretic devices allowed for external control of drug flow using a voltage source. The devices were tested in orthotopic mouse models of pancreatic and breast cancer as well as in a canine model for pharmacokinetic studies. Gemcitabine was delivered biweekly after suturing the devices onto orthotopic patient-derived pancreatic cancer xenograft tumors. They placed the counter electrode on the contralateral side of the tumor and applied a current of 2 mA for 10 min. By applying a current of 1 mA for 25 min, they demonstrated the delivery of cisplatin into human skin. These devices could be used in the future to treat many solid tumors.

Drug delivery by direct injection to the eye has been used to treat retinal diseases – such as glaucoma, age-related macular degeneration, diabetic retinopathy, and retinitis pigmentosa. As injection can be traumatic to the patient, Meng’s group developed a refillable drug delivery PDMS microfluidic device for the eye. They implanted the device under the conjunctiva (Fig. 9l). The device delivered the contents of its reservoir into the eye when it was manually pressurized (e.g. with a cotton swab) beyond the bursting pressure of the check valve^208,209^ (Fig. 9m, n). The reservoir featured posts to prevent stiction of the roof against the floor when it was collapsed by pressure application. The contents of the reservoir were re-filled with a sharp needle that punctured the PDMS, but the hole created by the needled was shown to self-seal.

Implantable microfluidic devices will eventually require the adoption of biocompatible materials in combination with wireless technologies. Choy’s group has fabricated implantable, biocompatible PMMA chips for the delivery of diclofenac sodium in PEG formulations; the devices were tested in rats^210^. Ryu and colleagues designed a biodegradable microfluidic device for the delivery of bupivacaine HCl (a local analgesic drug); the device was tested in vitro^211^. Langer’s group demonstrated wirelessly actuated implantable chips with multiple reservoirs for radio-controlled release of micro-doses of human parathyroid hormone fragment in patients^212^. The Takahata lab created an implantable wireless device capable of pumping micro-doses of dyes^213^. A team led by the Rogers lab demonstrated wireless optofluidic neural probes that combined ultrathin, soft microfluidic drug delivery with cellular-scale inorganic light-emitting diode arrays^214^ (Fig. 9o–r). In freely moving (untethered) wild-type mice, they used these minimally invasive probes to modify gene expression, to deliver peptide ligands, and to provide concurrent photostimulation and antagonist drug delivery in order to manipulate reward-related behaviors. Such probes promise to revolutionize our cellular-level understanding of many live, freely-behaving organisms, ranging from small animals to humans.

## Conclusions

Here we have reviewed how the field of microfluidics has contributed to advance the manipulation, sampling, and interrogation of live, intact tissues. We have seen how microfluidic technology is particularly well matched for addressing intact tissues because the availability of these tissues is often scarce (when they are from patient origin) and/or because multiplexing/automating the sampling and/or probing of the tissue using microchannel/microvalve delivery minimizes the overall cost of the experiment, optimizes mass transport, and (in the case of implantable devices) minimizes the chances of tissue damage. Future devices will incorporate more sensing capabilities so that the output is directly integrated within the platform; for ex., a tissue slice device might incorporate immunosensors for the measure of released cytokines in different portions of the slice, or a *Drosophila* embryo chip might incorporate homeostatic controls to keep pH and nutrients constant. These more sophisticated devices will allow for planning experiments of higher complexity. Presently, the biggest roadblock in building these integrated devices is the lack of a suitable, “common-denominator” fabrication technology – e.g. the electronics, the immunosensors, and the microfluidics are usually fabricated with three different techniques. The production of these multi-material chips will require new modular fabrication techniques – possibly based on digital manufacturing^215^ – and novel microfluidic designs that allow for monitoring the physiology and pathophysiology of the tissue.
